# Gut microbiota and cerebrovascular diseases: a Mendelian randomization study

**DOI:** 10.3389/fmicb.2023.1228815

**Published:** 2023-08-10

**Authors:** Hao Qin, Fan Yang, Pengfei Hao, Xianfeng Zhang

**Affiliations:** ^1^Department of Neurosurgery, The First Hospital of Jilin University, Changchun, China; ^2^Department of Anesthesiology, The First Hospital of Jilin University, Changchun, China; ^3^Department of Neurosurgery, Third Hospital of Shanxi Medical University, Shanxi Bethune Hospital, Shanxi Academy of Medical Sciences, Tongji Shanxi Hospital, Taiyuan, China

**Keywords:** gut microbiome, Mendelian randomization, stroke, intracranial aneurysms, causal effect

## Abstract

**Background:**

The causal relationship between gut microbiota and cerebrovascular disease remains unknown, despite several recent studies reporting an association between the two.

**Methods:**

To assess this relationship, we conducted a two-sample Mendelian randomization (MR) using summary statistics data from published genome-wide association studies (GWAS). This analysis allowed us to identify bacterial taxa that may affect cerebrovascular disease. Furthermore, we performed reverse MR to further analyze the significant bacterial taxa. Finally, we conducted a two-step MR analysis to examine the mediating role of metabolic factors [systolic blood pressure (SBP), type 2 diabetes (T2D), and body mass index (BMI)] in the association between gut microbiota and cerebrovascular disease. Additionally, a series of sensitivity analyses were carried out to validate the robustness of our findings.

**Results:**

Our results showed that a genetically predicted high abundance of family Porphyromonadaceae reduced the risk of intracranial aneurysms (IA). Moreover, using inverse variance weighted (IVW) estimates, we found a nominal causal relationship between seventeen gut microbiota and IA, as well as its subtypes. In the case of stroke and its subtypes, we observed a nominal causal relationship with thirteen, eleven, eleven, nine, and eight bacteria for AS, AIS, CES, LAS, and SVS, respectively. Reverse MR analysis showed no significant causal relationship between intracranial aneurysms and gut microbiota. However, we did find that genetically predicted any stroke (AS) and any ischemic stroke (AIS) reduced the abundance of family Clostridiaceae1 (OR: 0.74, 95% CI: 0.62–0.87, *p* = 3.39 × 10^−4^, and OR: 0.75, 95% CI: 0.66–0.87, *p* = 7.06 × 10^−5^, respectively). Furthermore, genetic prediction of AIS (OR: 0.87, 95% CI: 0.77–0.99, *p* = 3.05 × 10^−2^) was associated with a reduced abundance of the order Clostridiales. Moreover, genus Streptococcus exhibited effects on AS, AIS, and SVS which were mediated by T2D. Conversely, the association between genus *Eubacterium brachy* group and AIS was mediated by SBP. No significant heterogeneity of instrumental variables or horizontal pleiotropy was observed.

**Conclusion:**

This MR analysis indicates that there exists a beneficial or detrimental causal effect of gut microbiota composition on cerebrovascular disease. And SBP and T2D may play mediating role in this process.

## 1. Introduction

Cerebrovascular diseases, such as stroke, cerebral hemorrhage, and intracranial aneurysm (IA), impose a significant threat to human health and place a substantial burden on the healthcare system (GBD 2016 Causes of Death Collaborators, 2017). These diseases are associated with high mortality and disability rates. Stroke and cerebral hemorrhage, in particular, are prevalent and have a high incidence of mortality and disability (An et al., 2017). IA, which is a significant risk factor for cerebral hemorrhage, can result in severe neurological disorders in the event of rupture and bleeding (Tawk et al., 2021).

Gut microbiota, a group of microorganisms residing in the human gastrointestinal tract, depend on the gut environment to survive and perform various immune, metabolic, and endocrine activities (Sampson and Mazmanian, 2015). Numerous studies (Spence, 2018; English et al., 2021) have indicated that dietary and nutritional deficiencies, including a high intake of cholesterol-rich foods, can contribute to increased stroke mortality. Among them, gut microbiota may be the main regulators.

Gut microbiota may serve as key regulators in this process. For instance, Yin et al. (2015) discovered that patients experiencing stroke and transient ischemic attack had an enrichment of opportunistic gut-derived pathogens such as Enterobacter, Megasphaera, Oscillibacter, and Desulfovibrio, along with a reduction in beneficial genera like Bacteroides, Prevotella, and Faecalibacterium. Moreover, animal experiments have demonstrated a significant increase in the firmicutes/bacteroidetes ratio among aged ischemic stroke (IS) mice with imbalanced intestinal ecology. Feeding young mice with an increased firmicutes/bacteroidetes ratio through fecal transplant gavage (FTG) resulted in higher mortality after IS, while reducing the firmicutes/bacteroidetes ratio in aged mice improved their survival and aided in functional recovery (Spychala et al., 2018). Furthermore, significant changes in the gut microbiota, including a decrease in Hungatella hathewayi (Li et al., 2020) and an increase in campylobacter (Guo et al., 2021), have been found to impact the development, progression, and prognosis of IA.

Mendelian randomization in this context is a novel approach to exploring the causal relationship between gut microbiota and cerebrovascular diseases. Two-sample MR analyses were performed in this study using the summary GWAS data to assess the causal relationship between gut microbiota and cerebrovascular diseases. MR uses genetic variants to construct instrumental variables of exposure to estimate causal relationships between exposures and outcomes. Because the assignment of genotypes from parents to offspring is random, the association between genetic variation and outcome is not affected by common confounding factors. However, previous reports were subject to confounding factors such as age, socioeconomic factors, diet, and lifestyle, which may have biased the results. It is worth noting that the evidence so far has revealed associations between microbes and host physiology rather than proving causality.

## 2. Methods

### 2.1. GWAS data sources

All GWAS data sources were listed in Supplementary Table S1.

### 2.2. Gut microbiome data sources

SNPs related to the human gut microbiome composition were selected as instrumental variables (IVs) from a GWAS dataset of the international consortium MiBioGen (Kurilshikov et al., 2021). This was a multi-ethnic large-scale GWAS that coordinated 16S ribosomal RNA gene sequencing profiles and genotyping data from 18,340 participants from 24 cohorts from the United States, Canada, Israel, South Korea, Germany, Denmark, Netherlands, Belgium, Sweden, Finland, and the United Kingdom to explore the association between autosomal human genetic variants and the gut microbiome. A total of 211 taxa (131 genera, 35 families, 20 orders, 16 classes, and 9 phyla) were included. In our study, we finally included 194 taxa (119 genera, 30 families, 20 orders, 16 classes, and 9 phyla) after excluding unknown gut microbes.

### 2.3. Intracranial aneurysm data sources

Summary statistics data for intracranial aneurysm in individuals of European ancestry were obtained from a genome-wide association study of 23 cohorts comprising 79,429 individuals {7,495 cases [69% with ruptured intracranial aneurysm (RA), 28% with unruptured intracranial aneurysm (uIA), and 3.8% with unknown rupture status] and 71,934 controls} (Bakker et al., 2020).

### 2.4. Stroke data sources

The GWAS summary data for stroke were obtained from the MEGASTROKE consortium (Malik et al., 2018). This study included 29 studies including 40,585 cases and 406,111 controls. The MEGASTROKE consortium defined stroke as rapidly developing signs of focal (or global) disturbance of cerebral function, lasting >24 h or leading to death with no apparent cause other than that of vascular origin. The MEGASTROKE consortium divided stroke into as any stroke (AS), any ischemic stroke (AIS), large artery stroke (LAS), cardioembolic stroke (CES), small vessel stroke (SVS). We restricted the stroke population to European ancestry to minimize population stratification bias. A detailed description of the participants and study design of MEGASTROKE were provided in the original study (Malik et al., 2018).

### 2.5. Metabolic factors data sources

We downloaded the data of T2D, SBP, and BMI reported in the IEU Open GWAS project https://gwas.mrcieu.ac.uk/. Detailed information is provided in Supplementary Table S1.

### 2.6. SNP selection

To obtain unbiased estimates, instrumental variables (IVs) must satisfy three assumptions: (1) IVs are strongly associated with exposure factors; (2) IVs are not associated with any confounding factors affecting the exposure-outcome; (3) IVs can affect outcome only through exposure but not through other biological pathways.

The following selection criteria were used to choose the instrumental variables: (1) single nucleotide polymorphisms (SNPs) associated with each genus at the locus-wide significance threshold (*p* < 1.0 × 10^−5^) were selected as potential IVs (Sanna et al., 2019); (2) 1,000 Genomes project European samples data were used as the reference panel to calculate the linkage disequilibrium (LD) between the SNPs, and among those SNPs that had *R*^2^ < 0.001 (clumping window size = 10,000 kb), only the SNPs with the lowest *p*-values were retained; (3) SNPs with minor allele frequency (MAF) ≤ 0.01 were removed; and (4) when palindromic SNPs existed, the forward strand alleles were inferred using allele frequency information and it can avoid distortion of strand orientation or allele coding.

## 3. MR estimates

### 3.1. Statistical analysis

In this study, the main method used to examine the possible causal relationship between gut microbiota and cerebrovascular diseases was the inverse variance weighted (IVW) method. The IVW method utilized meta-analysis to combine Wald estimates for each SNP in order to obtain an overall estimate of the effect of gut microbiota on cerebrovascular disease. To ensure unbiased results, it was crucial to assume the absence of horizontal pleiotropy (Burgess et al., 2016). The MR-Egger regression is based on the assumption that instrument strength is not associated with direct effects (InSIDE), which makes it possible to assess the presence of pleiotropy using an intercept term. If the intercept term is equal to zero, it indicates that horizontal pleiotropy is not present and the results of MR-Egger regression are consistent with IVW (Bowden et al., 2015). The weighted median method allows for correct estimation of causality when up to 50% of instrumental variables are invalid (Burgess et al., 2016). If InSIDE assumptions are violated, weighted model estimates are found to have greater power to detect causal effects, less bias, and lower type I error rates than MR-Egger regression (Burgess et al., 2016).

### 3.2. Sensitivity analysis

To assess the causal relationship between the gut microbiota and cerebrovascular disease, several essential sensitivity analyses were conducted. Firstly, Cochran’s IVW Q statistic was used to test the heterogeneity of instrumental variables. Secondly, MR-Pleiotropy Residual Sum and Outlier (MR-PRESSO) analysis was performed to examine horizontal pleiotropy and eliminate SNPs with horizontal pleiotropic outliers, reducing the pleiotropy of causal effects (Verbanck et al., 2018). In our study, if there was significant horizontal pleiotropy in the MR-PRESSO global test, the outliers (*p*-value < 0.05) were removed and the remaining SNPs were re-analyzed in the IVW analysis. The MR-Egger regression intercept was also utilized to estimate the potential pleiotropy of SNPs, with a *p*-value > 0.05 indicating no horizontal pleiotropy (Bowden et al., 2015). Furthermore, to identify potentially heterogeneous SNPs, the “leave-one-out” analysis was performed by sequentially omitting each SNP. Additionally, to obtain a more rigorous interpretation of the causal relationship, reverse MR Analyses were conducted on bacteria that had been found to have a causal relationship with the outcome in the forward MR Analysis. The strength of individual instrumental variables was assessed using the formula: F = Beta^2^/SE^2^, where an F-statistic >10 indicated no significant weak instrument bias. Moreover, a Bonferroni correction was employed to account for multiple comparisons, with the significance threshold set at 0.05 divided by the number of bacteria under each attribute [genera: 0.05/119 (4.20 × 10^−4^), families: 0.05/30 (1.67 × 10^−3^), orders: 0.05/20 (2.50 × 10^−3^), classes: 0.05/16 (3.13 × 10^−3^), and phyla: 0.05/9 (5.56 × 10^−3^)]. Lastly, a reverse causality analysis was conducted to examine the reverse causal association, with a *p*-value between 0.05 and the corrected value being considered to have a nominal causal effect.

## 4. Mediation Mendelian randomization

A two-step Mendelian randomization analysis was conducted to explore the mediating relationship between metabolic factors (SBP, T2D, and BMI) and the association between gut microbiota and cerebrovascular disease. First, causal relationships between gut microbiota and the metabolic factors were explored separately using Mendelian randomization. Second, the causal relationship between the metabolic factors and cerebrovascular disease was further examined. To determine the mediating effect of metabolic factors on the causal relationship between gut microbiota and cerebrovascular disease, the total effect was defined as β1. The causal effect between gut microbiota and metabolic factors was defined as β2, while the causal effect between metabolic factors and cerebrovascular disease was defined as β3. β2 × β3 represented the mediating effect, and β2 × β3/β1 represented the percentage of the mediating effect.

All statistical analyses were performed using R version 4.2.2 (R Foundation for Statistical Computing, Vienna, Austria). MR analyses were performed using the TwosampleMR (version 0.5.6) (Hemani et al., 2017), MR-PRESSO (Verbanck et al., 2018) R packages.

## 5. Results

### 5.1. Genetic instruments for gut microbiome

There were 194 bacterial traits containing five biological levels (i.e., phylum, class, order family, and genus) in our study. The detailed information of the final SNPs for each bacterial trait were shown in Supplementary Tables S2, S3. The *F* values >10, indicating there was no weak instrument bias.

### 5.2. Causal effects of gut microbiota on intracranial aneurysms

**Figure 1:**
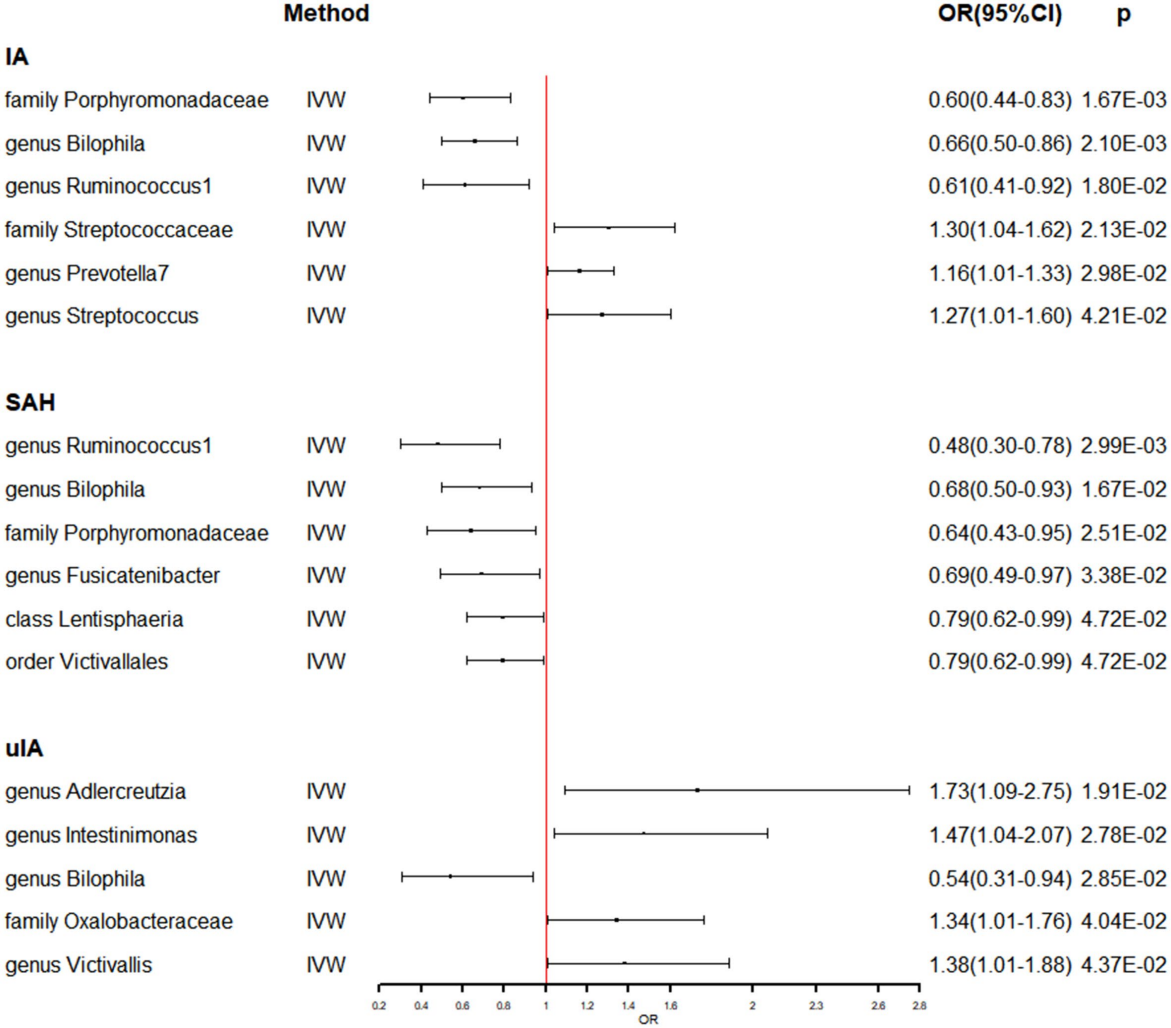
The association between genetically determined gut microbiome and the risk of IA in the primary results. OR, odds ratios; CI, confidence interval; IA, intracranial aneurysm; SAH, subarachnoid hemorrhage; uIA, unruptured intracranial aneurysm.

Supplementary Table S4 shows that the majority of gut microbiomes were not related to IA. However, among 194 gut bacterial features, family Porphyromonadaceae was predicted to have a strong causal relationship with IA, with an odds ratio (OR) of 0.60 and a 95% confidence interval (CI) of 0.44–0.83 (*p* = 1.67 × 10^−3^) (Table 1 and Figure 1). Additionally, two gut microbes, genus Bilophila (OR: 0.66, 95% CI: 0.50–0.86) and genus Ruminococcus1 (OR: 0.61, 95% CI: 0.41–0.92), were found to reduce the risk of IA (Table 1 and Figure 1). On the other hand, three gut microbiota, family Streptococcaceae (OR: 1.30, 95% CI: 1.04–1.62), genus Prevotella7 (OR: 1.16, 95% CI: 1.01–1.33), and genus Streptococcus (OR: 1.27, 95% CI: 1.01–1.60), were associated with a high risk of IA (Table 1 and Figure 1). In the IA subtype analysis, six gut microbial abundances were associated with a low risk of subarachnoid hemorrhage (SAH): genus Ruminococcus1 (OR: 0.48, 95% CI: 0.30–0.78), genus Bilophila (OR: 0.68, 95% CI: 0.50–0.93), family Porphyromonadaceae (OR: 0.64, 95% CI: 0.43–0.95), genus Fusicatenibacter (OR: 0.69, 95% CI: 0.49–0.67), class Lentisphaeria (OR: 0.79, 95% CI: 0.62–0.99), and order Victivallales (OR: 0.79, 95% CI: 0.62–0.99) (Table 1 and Figure 1). On the other hand, four gut microbial abundances were associated with a high risk of uIA: genus Adlercreutzia (OR: 1.73, 95% CI: 1.09–2.75), genus Intestinimonas (OR: 1.47, 95% CI: 1.04–2.07), family Oxalobacteraceae (OR: 1.34, 95% CI: 1.01–1.76), and genus Victivallis (OR: 1.38, 95% CI: 1.01–1.88) (Table 1 and Figure 1). Furthermore, genus Bilophila was found to reduce the risk of uIA, with an OR of 0.54 and a 95% CI of 0.31–0.94 (Table 1 and Figure 1). There was no evidence of heterogeneity among the IVs (Table 1 and Supplementary Table S6), and the MR-Egger regression intercepts did not deviate from zero, indicating no evidence of horizontal pleiotropy (*p* > 0.05 for all intercepts, Table 1 and Supplementary Table S6). Additionally, the leave-one-out analyses revealed that none of the identified causal associations were driven by any individual IV.

**Table 1:** Positive MR results of causal links between gut microbiome and intracranial aneurysms.

Exposures	Outcomes	Method	OR(95%CI)	*p*	P_Q	Egger intercept	P_intercept	MR-PRESSO
family Porphyromonadaceae	IA	IVW	0.60(0.44–0.83)	1.67E-03	7.03E-01	8.63E-04	9.86E-01	–
genus Bilophila	IA	IVW	0.66(0.50–0.86)	2.10E-03	6.07E-01	2.00E-02	7.51E-01	–
genus Ruminococcus1	IA	IVW	0.61(0.41–0.92)	1.80E-02	6.69E-01	-4.31E-02	4.17E-01	–
family Streptococcaceae	IA	IVW	1.30(1.04–1.62)	2.13E-02	4.64E-01	-7.08E-03	8.45E-01	–
genus Prevotella7	IA	IVW	1.16(1.01–1.33)	2.98E-02	5.45E-01	−8.75E-02	2.88E-01	–
genus Streptococcus	IA	IVW	1.27(1.01–1.60)	4.21E-02	9.04E-01	−1.75E-02	5.75E-01	–
genus Ruminococcus1	SAH	IVW	0.48(0.30–0.78)	2.99E-03	9.64E-01	−1.56E-02	7.86E-01	–
genus Bilophila	SAH	IVW	0.68(0.50–0.93)	1.67E-02	5.40E-01	4.72E-03	9.48E-01	–
family Porphyromonadaceae	SAH	IVW	0.64(0.43–0.95)	2.51E-02	3.33E-01	4.14E-02	5.32E-01	–
genus Fusicatenibacter	SAH	IVW	0.69(0.49–0.97)	3.38E-02	9.50E-01	−1.00E-02	8.58E-01	–
class Lentisphaeria	SAH	IVW	0.79(0.62–0.99)	4.72E-02	2.76E-01	−2.06E-02	7.55E-01	–
order Victivallales	SAH	IVW	0.79(0.62–0.99)	4.72E-02	2.76E-01	−2.06E-02	7.55E-01	–
genus Adlercreutzia	uIA	IVW	1.73(1.09–2.75)	1.91E-02	4.94E-01	−9.89E-02	3.90E-01	–
genus Intestinimonas	uIA	IVW	1.47(1.04–2.07)	2.78E-02	9.12E-01	−1.12E-02	7.87E-01	–
genus Bilophila	uIA	IVW	0.54(0.31–0.94)	2.85E-02	2.33E-01	6.66E-02	6.35E-01	–
family Oxalobacteraceae	uIA	IVW	1.34(1.01–1.76)	4.04E-02	5.81E-01	1.32E-01	1.28E-01	–
genus Victivallis	uIA	IVW	1.38(1.01–1.88)	4.37E-02	4.12E-01	5.46E-02	8.43E-01	–

OR, odds ratio; CI, confidence interval; MRPRESSO, MR-Pleiotropy Residual Sum and Outlier; IA, intracranial aneurysms; SAH, subarachnoid hemorrhage; uIA, unruptured intracranial aneurysm; IVW, Inverse variance weighted.

### 5.3. Causal effects of gut microbiota on stroke

**Figure 2:**
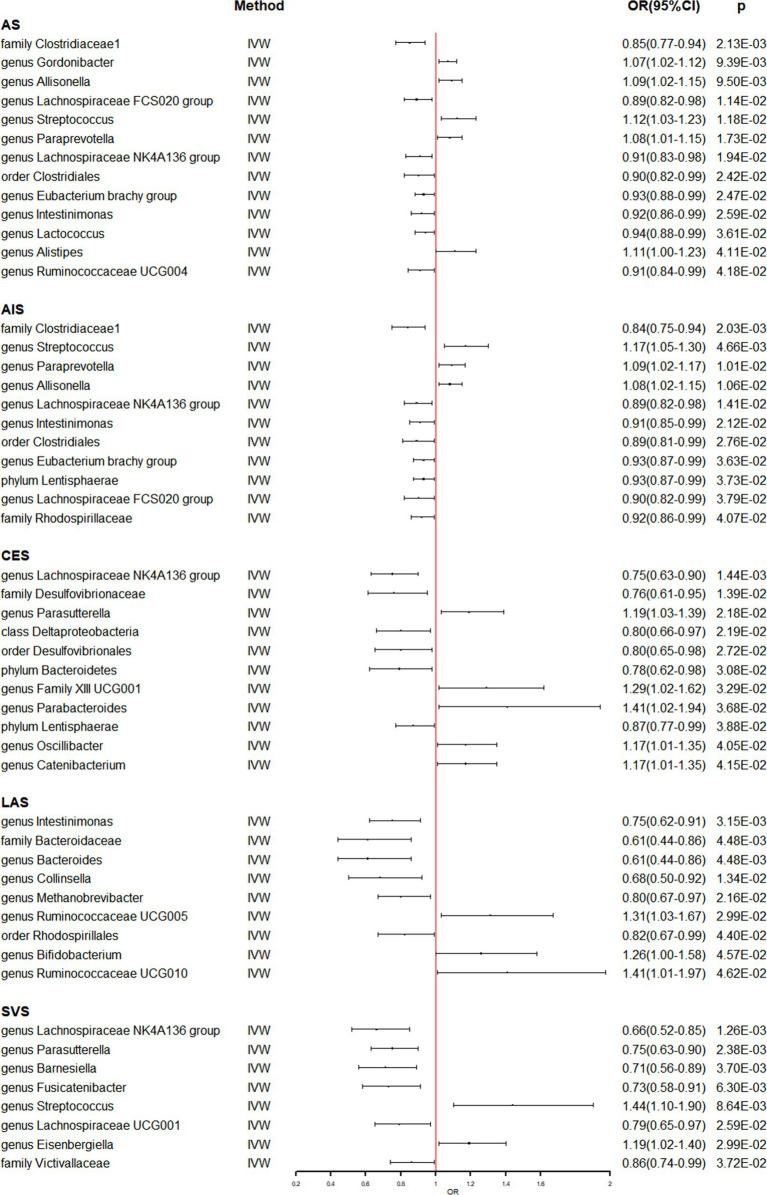
The association between genetically determined gut microbiome and the risk of stroke and its subtypes in the primary results. OR, odds ratios; CI, confidence interval; AS, any stroke; AIS, any ischemic stroke; LAS, large artery stroke; CES, cardioembolic stroke; SVS, small vessel stroke.

Meanwhile, the identical approaches were utilized to explore the causal effect of gut microbiome on stroke and its subtypes (Supplementary Table S5). Table 2 and Figure 2 present the potential causal relationships between specific gut microbes and stroke, as well as its subtypes. In the overall analysis of stroke, various gut microbes were found to have potential causal relationships with AS and AIS. These include nine gut microbes such as family Clostridiaceae1, genus Allisonella, genus Lachnospiraceae FCS020 group, genus Streptococcus, genus Paraprevotella, genus Lachnospiraceae NK4A136 group, order Clostridiales, genus *Eubacterium brachy* group, and genus Intestinimonas. Similarly, the analysis revealed potential causal associations between certain gut microbes and the risk of AS. These included genus Gordonibacter (OR: 1.07, 95% CI: 1.02–1.12), genus Lactococcus (OR: 0.94, 95% CI: 0.88–0.99), genus Alistipes (OR: 1.11, 95% CI: 1.00–1.23), and genus Ruminococcaceae UCG004 (OR: 0.91, 95% CI: 0.84–0.99) (Table 2 and Figure 2). On the other hand, Phylum Lentisphaerae (OR: 0.93, 95% CI: 0.87–0.99) and family Rhodospirillaceae (OR: 0.92, 95% CI: 0.86–0.99) were found to have potential causal relationships with AIS (Table 2 and Figure 2). The analysis also focused on the subtypes of stroke. It was discovered that six gut microbes, namely genus Lachnospiraceae NK4A136 group (OR: 0.75, 95% CI: 0.63–0.90), family Desulfovibrionaceae (OR: 0.76, 95% CI: 0.61–0.95), class Deltaproteobacteria (OR: 0.80, 95% CI: 0.66–0.97), order Desulfovibrionales (OR: 0.80, 95% CI: 0.65–0.98), phylum Bacteroidetes (OR: 0.78, 95% CI: 0.62–0.98), and phylum Lentisphaerae (OR: 0.87, 95% CI: 0.77–0.99) were associated with a reduced risk of CES. In contrast, five gut microbes, including genus Parasutterella (OR: 1.19, 95% CI: 1.03–1.39), genus Family XIII UCG001 (OR: 1.29, 95% CI: 1.02–1.62), genus Parabacteroides (OR: 1.41, 95% CI: 1.02–1.94), genus Oscillibacter (OR: 1.17, 95% CI: 1.01–1.35), and genus Catenibacterium (OR: 1.17, 95% CI: 1.01–1.35) were associated with an increased risk of CES (Table 2 and Figure 2). Likewise, six gut microbes (genus Intestinimonas: OR: 0.75, 95% CI: 0.62–0.91; family Bacteroidaceae: OR: 0.61, 95% CI: 0.44–0.86; genus Bacteroides: OR: 0.61, 95% CI: 0.44–0.86; genus Collinsella: OR: 0.68, 95% CI: 0.50–0.92; genus Methanobrevibacter: OR: 0.80, 95% CI: 0.67–0.97; order Rhodospirillales: OR: 0.82, 95% CI: 0.67–0.99) were associated with reduced risk of LAS, while three gut microbes (genus Ruminococcaceae UCG005: OR: 1.31, 95% CI: 1.03–1.67; genus Bifidobacterium: OR: 1.26, 95% CI: 1.00–1.58; genus Ruminococcaceae UCG010: OR: 1.41, 95% CI: 1.01–1.97) were associated with increased risk of LAS (Table 2 and Figure 2). In the analysis of SVS, six gut microbes (genus Lachnospiraceae NK4A136 group: OR: 0.66, 95% CI: 0.52–0.85; genus Parasutterella: OR: 0.75, 95% CI: 0.63–0.90; genus Barnesiella: OR: 0.71, 95% CI: 0.56–0.89; genus Fusicatenibacter: OR: 0.73, 95% CI: 0.58–0.91; genus Lachnospiraceae UCG001: OR: 0.79, 95% CI: 0.65–0.97; family Victivallaceae: OR: 0.86, 95% CI: 0.74–0.99) were associated with reduced risk of SVS. Conversely, the presence of two gut microbes (genus Streptococcus: OR: 1.44, 95% CI: 1.10–1.90; genus Eisenbergiella: OR: 1.19, 95% CI: 1.02–1.40) were associated with increased risk of SVS (Table 2 and Figure 2). Importantly, no evidence of heterogeneity and horizontal pleiotropy was observed among the instrumental variables (IVs), indicating the reliability of the findings (Table 2 and Supplementary Table S6). Additionally, the leave-one-out analyses confirmed that no individual IV significantly influenced the identified causal associations.

**Table 2:** Positive MR results of causal links between gut microbiome and stroke.

Exposures	Outcomes	Method	OR(95%CI)	*p*	P_Q	Egger intercept	P_intercept	MR-PRESSO
family Clostridiaceae1	AS	IVW	0.85(0.77–0.94)	2.13E-03	6.00E-01	1.28E-02	3.01E-01	–
genus Gordonibacter	AS	IVW	1.07(1.02–1.12)	9.39E-03	7.70E-01	1.16E-02	4.98E-01	–
genus Allisonella	AS	IVW	1.09(1.02–1.15)	9.50E-03	2.80E-01	4.51E-02	1.43E-01	–
genus Lachnospiraceae FCS020 group	AS	IVW	0.89(0.82–0.98)	1.14E-02	9.49E-01	1.27E-05	9.99E-01	–
genus Streptococcus	AS	IVW	1.12(1.03–1.23)	1.18E-02	4.64E-01	1.92E-02	1.47E-01	–
genus Paraprevotella	AS	IVW	1.08(1.01–1.15)	1.73E-02	5.66E-01	−1.87E-04	9.87E-01	–
genus Lachnospiraceae NK4A136 group	AS	IVW	0.91(0.83–0.98)	1.94E-02	5.99E-01	−1.22E-02	7.31E-02	–
order Clostridiales	AS	IVW	0.90(0.82–0.99)	2.42E-02	9.20E-01	−1.08E-02	2.04E-01	–
genus *Eubacterium brachy* group	AS	IVW	0.93(0.88–0.99)	2.47E-02	4.16E-01	3.64E-02	7.46E-02	–
genus Intestinimonas	AS	IVW	0.92(0.86–0.99)	2.59E-02	5.59E-01	2.81E-03	7.36E-01	–
genus Lactococcus	AS	IVW	0.94(0.88–0.99)	3.61E-02	5.84E-01	1.79E-02	3.51E-01	–
genus Alistipes	AS	IVW	1.11(1.00–1.23)	4.11E-02	8.66E-01	7.08E-03	6.41E-01	–
genus Ruminococcaceae UCG004	AS	IVW	0.91(0.84–0.99)	4.18E-02	4.08E-01	−9.08E-03	6.71E-01	–
family Clostridiaceae1	AIS	IVW	0.84(0.75–0.94)	2.03E-03	8.34E-01	1.28E-02	3.44E-01	–
genus Streptococcus	AIS	IVW	1.17(1.05–1.30)	4.66E-03	2.68E-01	1.61E-02	2.89E-01	–
genus Paraprevotella	AIS	IVW	1.09(1.02–1.17)	1.01E-02	4.29E-01	3.98E-03	7.68E-01	–
genus Allisonella	AIS	IVW	1.08(1.02–1.15)	1.06E-02	4.38E-01	3.37E-02	2.91E-01	–
genus Lachnospiraceae NK4A136 group	AIS	IVW	0.89(0.82–0.98)	1.41E-02	5.21E-01	−1.12E-02	1.22E-01	–
genus Intestinimonas	AIS	IVW	0.91(0.85–0.99)	2.12E-02	4.32E-01	6.57E-03	4.84E-01	–
order Clostridiales	AIS	IVW	0.89(0.81–0.99)	2.76E-02	8.60E-01	−1.11E-02	2.31E-01	–
genus *Eubacterium brachy* group	AIS	IVW	0.93(0.87–0.99)	3.63E-02	6.95E-01	3.14E-02	1.40E-01	–
phylum Lentisphaerae	AIS	IVW	0.93(0.87–0.99)	3.73E-02	3.89E-01	−1.53E-03	9.42E-01	–
genus Lachnospiraceae FCS020 group	AIS	IVW	0.90(0.82–0.99)	3.79E-02	6.49E-01	−5.58E-03	5.51E-01	–
family Rhodospirillaceae	AIS	IVW	0.92(0.86–0.99)	4.07E-02	9.50E-01	−1.09E-02	6.25E-01	–
genus Lachnospiraceae NK4A136 group	CES	IVW	0.75(0.63–0.90)	1.44E-03	6.59E-01	9.88E-04	9.42E-01	–
family Desulfovibrionaceae	CES	IVW	0.76(0.61–0.95)	1.39E-02	3.69E-01	5.39E-03	8.19E-01	–
genus Parasutterella	CES	IVW	1.19(1.03–1.39)	2.18E-02	7.71E-01	1.20E-02	5.13E-01	–
class Deltaproteobacteria	CES	IVW	0.80(0.66–0.97)	2.19E-02	4.75E-01	1.37E-02	5.01E-01	–
order Desulfovibrionales	CES	IVW	0.80(0.65–0.98)	2.72E-02	3.93E-01	1.35E-02	5.20E-01	–
phylum Bacteroidetes	CES	IVW	0.78(0.62–0.98)	3.08E-02	3.13E-01	4.19E-03	8.37E-01	–
genus Family XIII UCG001	CES	IVW	1.29(1.02–1.62)	3.29E-02	4.01E-01	2.25E-02	4.70E-01	–
genus Parabacteroides	CES	IVW	1.41(1.02–1.94)	3.68E-02	2.98E-01	9.54E-03	8.71E-01	–
phylum Lentisphaerae	CES	IVW	0.87(0.77–0.99)	3.88E-02	8.71E-01	−4.69E-03	9.00E-01	–
genus Oscillibacter	CES	IVW	1.17(1.01–1.35)	4.05E-02	6.58E-01	1.93E-02	4.71E-01	–
genus Catenibacterium	CES	IVW	1.17(1.01–1.35)	4.15E-02	5.15E-01	1.19E-01	2.83E-01	–
genus Intestinimonas	LAS	IVW	0.75(0.62–0.91)	3.15E-03	4.93E-01	9.94E-03	6.64E-01	–
family Bacteroidaceae	LAS	IVW	0.61(0.44–0.86)	4.48E-03	5.19E-01	−4.25E-02	4.83E-01	–
genus Bacteroides	LAS	IVW	0.61(0.44–0.86)	4.48E-03	5.19E-01	−4.25E-02	4.83E-01	–
genus Collinsella	LAS	IVW	0.68(0.50–0.92)	1.34E-02	4.63E-01	2.21E-02	6.17E-01	–
genus Methanobrevibacter	LAS	IVW	0.80(0.67–0.97)	2.16E-02	7.23E-01	3.56E-02	5.24E-01	–
genus Ruminococcaceae UCG005	LAS	IVW	1.31(1.03–1.67)	2.99E-02	4.27E-01	−6.78E-03	8.25E-01	–
order Rhodospirillales	LAS	IVW	0.82(0.67–0.99)	4.40E-02	7.68E-01	1.47E-02	7.86E-01	–
genus Bifidobacterium	LAS	IVW	1.26(1.00–1.58)	4.57E-02	4.48E-01	−2.26E-03	9.27E-01	–
genus Ruminococcaceae UCG010	LAS	IVW	1.41(1.01–1.97)	4.62E-02	9.02E-01	2.13E-02	6.12E-01	–
genus Lachnospiraceae NK4A136 group	SVS	IVW	0.66(0.52–0.85)	1.26E-03	1.33E-01	3.77E-03	8.49E-01	–
genus Parasutterella	SVS	IVW	0.75(0.63–0.90)	2.38E-03	4.17E-01	−8.45E-03	7.15E-01	–
genus Barnesiella	SVS	IVW	0.71(0.56–0.89)	3.70E-03	6.01E-01	9.06E-03	7.75E-01	–
genus Fusicatenibacter	SVS	IVW	0.73(0.58–0.91)	6.30E-03	3.57E-01	6.37E-03	8.44E-01	–
genus Streptococcus	SVS	IVW	1.44(1.10–1.90)	8.64E-03	1.06E-01	7.60E-03	8.51E-01	–
genus Lachnospiraceae UCG001	SVS	IVW	0.79(0.65–0.97)	2.59E-02	9.48E-01	−1.15E-02	7.90E-01	–
genus Eisenbergiella	SVS	IVW	1.19(1.02–1.40)	2.99E-02	8.20E-01	−9.19E-02	1.91E-01	–
family Victivallaceae	SVS	IVW	0.86(0.74–0.99)	3.72E-02	1.78E-01	9.34E-03	8.57E-01	–

OR, odds ratio; CI, confidence interval; MRPRESSO, MR-Pleiotropy Residual Sum and Outlier; AS, any stroke; AIS, any ischemic stroke; CES, cardioembolic stroke; LAS, large artery stroke; SVS, small vessel stroke; IVW, Inverse variance weighted.

### 5.4. Reverse Mendelian randomization analysis

**Figure 3:**
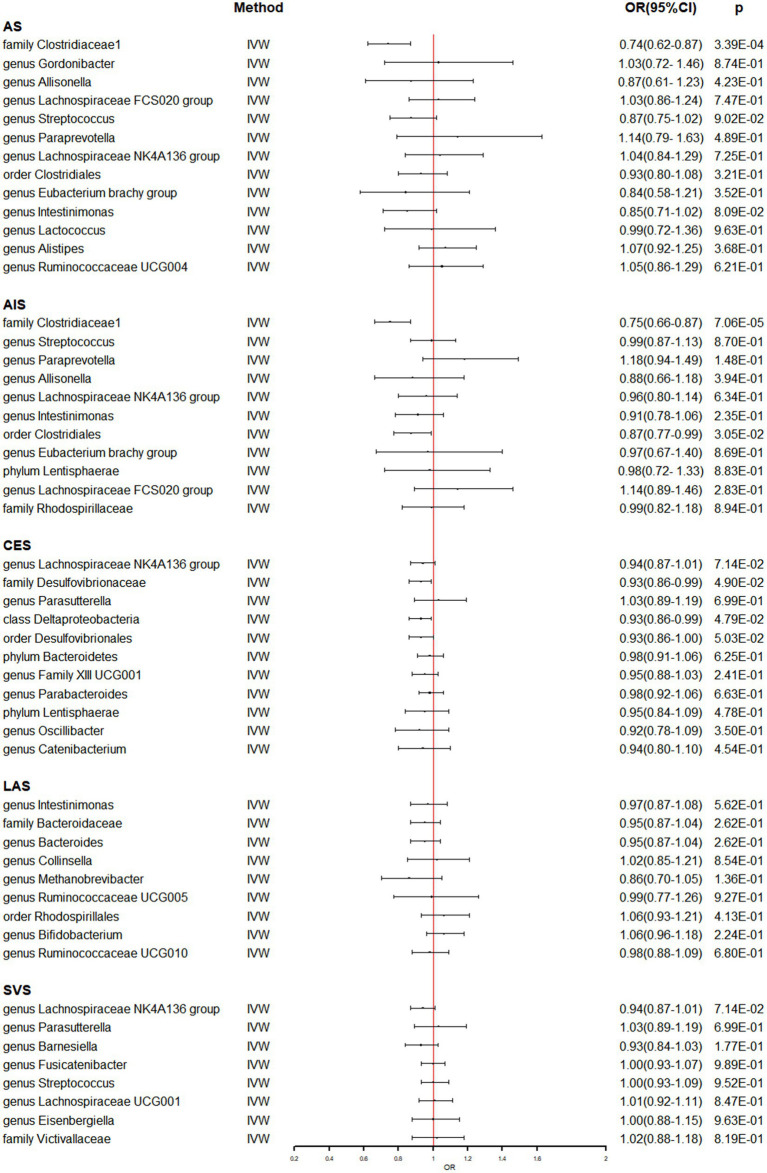
The association between genetically determined stroke and gut microbiome in the primary results. OR, odds ratios; CI, confidence interval; AS, any stroke; AIS, any ischemic stroke; LAS, large artery stroke; CES, cardioembolic stroke; SVS, small vessel stroke.

**Figure 4:**
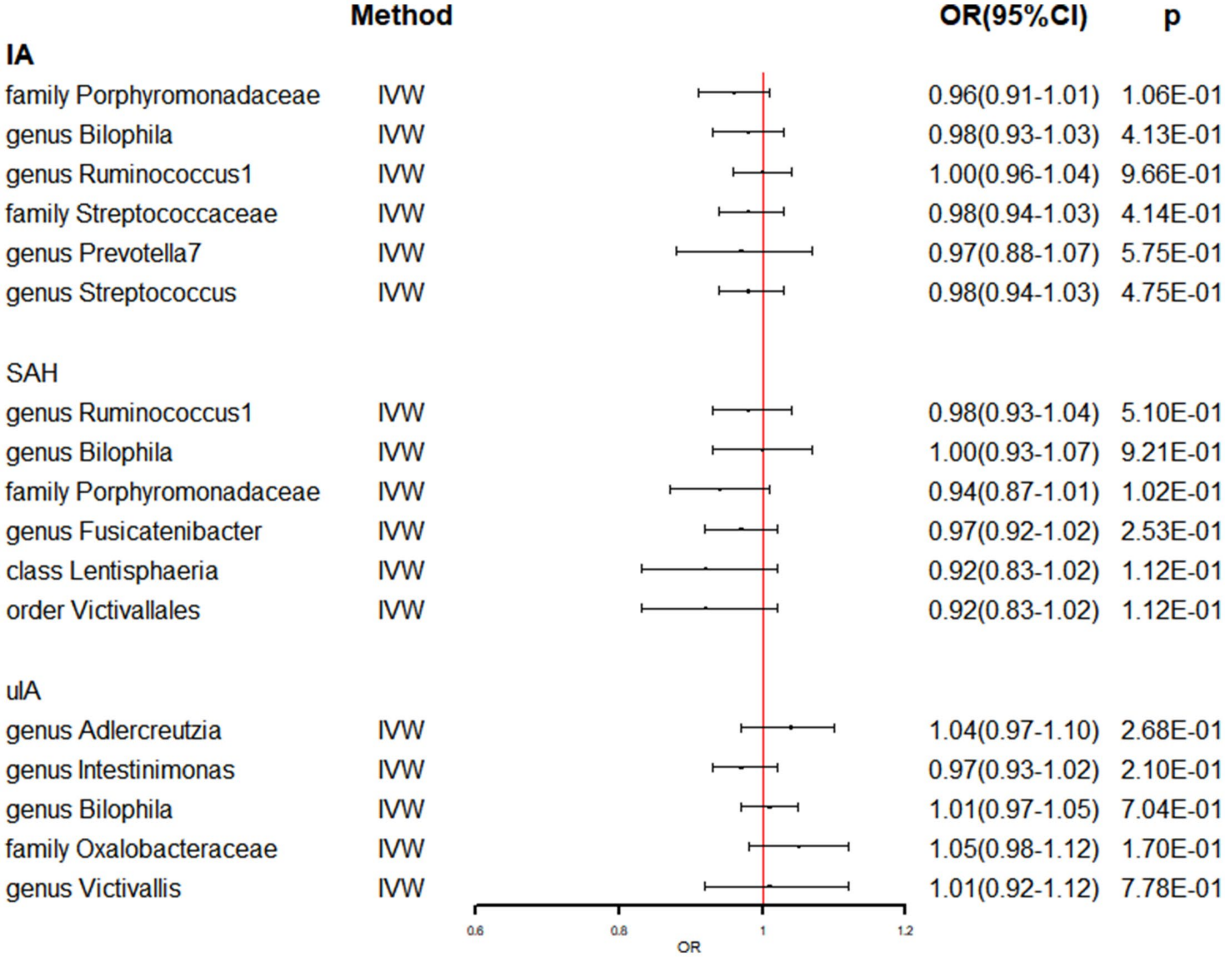
The association between genetically determined IA and gut microbiome in the primary results. OR, odds ratios; CI, confidence interval; IA, intracranial aneurysm; SAH, subarachnoid hemorrhage; uIA, unruptured intracranial aneurysm.

In Supplementary Table S7, the detailed information of the final SNPs for IA and stroke was provided. The reverse MR analysis indicated that genetically predicted AS and AIS had a significant impact on the abundance of family Clostridiaceae1 (OR: 0.74, 95% CI: 0.62–0.87, *p* = 3.39 × 10^−4^) (Table 3 and Figure 3). Additionally, genetic prediction of AIS showed a reduced abundance of order Clostridiales (OR: 0.87, 95% CI: 0.77–0.99, *p* = 3.05 × 10^−2^) (Table 3 and Figure 3). Furthermore, a nominal association was found between CES and family Desulfovibrionaceae (OR: 0.93, 95% CI: 0.86–0.99, *p* = 4.90 × 10^−2^) as well as class Deltaproteobacteria (OR: 0.93, 95% CI: 0.86–0.99, *p* = 4.79 × 10^−2^) (Table 3 and Figure 3). However, no significant causal effect of intracranial aneurysm on gut microbiota was observed (Table 4 and Figure 4), and there was no evidence of heterogeneity and horizontal pleiotropy (Tables 3, 4). Additionally, the leave-one-out analyses revealed that none of the identified causal associations were driven by any individual IV.

**Table 3:** Reverse MR results of causal links between stroke and gut microbiome.

Outcomes	Exposures	Method	OR(95%CI)	*p*	P_Q	Egger intercept	P_intercept	MR-PRESSO
family Clostridiaceae1	AS	IVW	0.74(0.62–0.87)	3.39E-04	7.74E-01	2.92E-02	5.15E-01	–
genus Gordonibacter	AS	IVW	1.03(0.72–1.46)	8.74E-01	3.34E-01	−1.47E-01	1.43E-01	–
genus Allisonella	AS	IVW	0.87(0.61–1.23)	4.23E-01	9.55E-01	−1.39E-02	8.79E-01	–
genus Lachnospiraceae FCS020 group	AS	IVW	1.03(0.86–1.24)	7.47E-01	3.00E-01	4.96E-02	3.33E-01	–
genus Streptococcus	AS	IVW	0.87(0.75–1.02)	9.02E-02	9.74E-01	−5.54E-03	8.92E-01	–
genus Paraprevotella	AS	IVW	1.14(0.79–1.63)	4.89E-01	4.02E-02	−7.93E-02	4.26E-01	–
genus Lachnospiraceae NK4A136 group	AS	IVW	1.04(0.84–1.29)	7.25E-01	7.07E-02	2.89E-02	6.42E-01	–
order Clostridiales	AS	IVW	0.93(0.80–1.08)	3.21E-01	9.17E-01	1.53E-02	6.93E-01	–
genus *Eubacterium brachy* group	AS	IVW	0.84(0.58–1.21)	3.52E-01	2.35E-01	−1.90E-02	8.59E-01	–
genus Intestinimonas	AS	IVW	0.85(0.71–1.02)	8.09E-02	6.35E-01	8.40E-03	8.59E-01	–
genus Lactococcus	AS	IVW	0.99(0.72–1.36)	9.63E-01	9.44E-01	3.85E-02	6.42E-01	–
genus Alistipes	AS	IVW	1.07(0.92–1.25)	3.68E-01	4.87E-01	−1.23E-02	7.64E-01	–
genus Ruminococcaceae UCG004	AS	IVW	1.05(0.86–1.29)	6.21E-01	4.62E-01	5.92E-02	3.04E-01	–
family Clostridiaceae1	AIS	IVW	0.75(0.66–0.87)	7.06E-05	8.39E-01	5.12E-02	3.53E-01	–
genus Streptococcus	AIS	IVW	0.99(0.87–1.13)	8.70E-01	6.54E-01	2.90E-02	5.62E-01	–
genus Paraprevotella	AIS	IVW	1.18(0.94–1.49)	1.48E-01	2.37E-01	−1.76E-02	8.50E-01	–
genus Allisonella	AIS	IVW	0.88(0.66–1.18)	3.94E-01	6.65E-01	6.68E-02	5.52E-01	–
genus Lachnospiraceae NK4A136 group	AIS	IVW	0.96(0.80–1.14)	6.34E-01	7.02E-02	1.77E-02	8.08E-01	–
genus Intestinimonas	AIS	IVW	0.91(0.78–1.06)	2.35E-01	7.49E-01	3.21E-02	5.81E-01	–
order Clostridiales	AIS	IVW	0.87(0.77–0.99)	3.05E-02	7.49E-01	1.26E-02	7.87E-01	–
genus *Eubacterium brachy* group	AIS	IVW	0.97(0.67–1.40)	8.69E-01	7.16E-02	1.92E-01	1.58E-01	–
phylum Lentisphaerae	AIS	IVW	0.98(0.72–1.33)	8.83E-01	1.32E-01	−9.60E-02	4.41E-01	–
genus Lachnospiraceae FCS020 group	AIS	IVW	1.14(0.89–1.46)	2.83E-01	6.30E-03	5.17E-02	6.06E-01	–
family Rhodospirillaceae	AIS	IVW	0.99(0.82–1.18)	8.94E-01	9.64E-01	−1.58E-02	8.18E-01	–
genus Lachnospiraceae NK4A136 group	CES	IVW	0.94(0.87–1.01)	7.14E-02	9.31E-01	−6.43E-03	7.79E-01	–
family Desulfovibrionaceae	CES	IVW	0.93(0.86–0.99)	4.90E-02	7.41E-01	−4.09E-03	8.64E-01	–
genus Parasutterella	CES	IVW	1.03(0.89–1.19)	6.99E-01	5.89E-02	−2.43E-02	6.89E-01	–
class Deltaproteobacteria	CES	IVW	0.93(0.86–0.99)	4.79E-02	6.77E-01	−5.26E-03	8.27E-01	–
order Desulfovibrionales	CES	IVW	0.93(0.86–1.00)	5.03E-02	7.35E-01	−5.82E-03	8.10E-01	–
phylum Bacteroidetes	CES	IVW	0.98(0.91–1.06)	6.25E-01	2.91E-01	8.34E-03	8.00E-01	–
genus Family XIII UCG001	CES	IVW	0.95(0.88–1.03)	2.41E-01	7.61E-01	−1.53E-02	5.95E-01	–
genus Parabacteroides	CES	IVW	0.98(0.92–1.06)	6.63E-01	7.29E-01	−9.77E-03	6.79E-01	–
phylum Lentisphaerae	CES	IVW	0.95(0.84–1.09)	4.78E-01	8.43E-01	−1.95E-02	6.66E-01	–
genus Oscillibacter	CES	IVW	0.92(0.78–1.09)	3.50E-01	5.85E-02	5.88E-02	2.53E-01	–
genus Catenibacterium	CES	IVW	0.94(0.80–1.10)	4.54E-01	4.50E-01	−2.59E-02	6.64E-01	–
genus Intestinimonas	LAS	IVW	0.97(0.87–1.08)	5.62E-01	–	–	–	–
family Bacteroidaceae	LAS	IVW	0.95(0.87–1.04)	2.62E-01	–	–	–	–
genus Bacteroides	LAS	IVW	0.95(0.87–1.04)	2.62E-01	–	–	–	–
genus Collinsella	LAS	IVW	1.02(0.85–1.21)	8.54E-01	–	–	–	–
genus Methanobrevibacter	LAS	IVW	0.86(0.70–1.05)	1.36E-01	–	–	–	–
genus Ruminococcaceae UCG005	LAS	IVW	0.99(0.77–1.26)	9.27E-01	–	–	–	–
order Rhodospirillales	LAS	IVW	1.06(0.93–1.21)	4.13E-01	–	–	–	–
genus Bifidobacterium	LAS	IVW	1.06(0.96–1.18)	2.24E-01	–	–	–	–
genus Ruminococcaceae UCG010	LAS	IVW	0.98(0.88–1.09)	6.80E-01	–	–	–	–
genus Lachnospiraceae NK4A136 group	SVS	IVW	0.94(0.87–1.01)	7.14E-02	9.31E-01	−6.43E-03	7.79E-01	–
genus Parasutterella	SVS	IVW	1.03(0.89–1.19)	6.99E-01	5.89E-02	−2.43E-02	6.89E-01	–
genus Barnesiella	SVS	IVW	0.93(0.84–1.03)	1.77E-01	1.46E-01	−1.30E-02	7.80E-01	–
genus Fusicatenibacter	SVS	IVW	1.00(0.93–1.07)	9.89E-01	7.98E-01	−1.00E-02	6.71E-01	–
genus Streptococcus	SVS	IVW	1.00(0.93–1.09)	9.52E-01	2.87E-01	−2.17E-02	4.58E-01	–
genus Lachnospiraceae UCG001	SVS	IVW	1.01(0.92–1.11)	8.47E-01	9.07E-01	−1.99E-03	9.45E-01	–
genus Eisenbergiella	SVS	IVW	1.00(0.88–1.15)	9.63E-01	3.05E-01	2.84E-02	5.96E-01	–
family Victivallaceae	SVS	IVW	1.02(0.88–1.18)	8.19E-01	9.18E-01	8.73E-03	8.53E-01	–

OR, odds ratio; CI, confidence interval; MRPRESSO, MR-Pleiotropy Residual Sum and Outlier; AS, any stroke; AIS, any ischemic stroke; CES, cardioembolic stroke; LAS, large artery stroke; SVS, small vessel stroke; IVW, Inverse variance weighted.

**Table 4:** Reverse MR results of causal links between intracranial aneurysms and gut microbiome.

Outcomes	Exposures	Method	OR(95%CI)	*p*	P_Q	Egger intercept	P_intercept	MR-PRESSO
family Porphyromonadaceae	IA	IVW	0.96(0.91–1.01)	1.06E-01	1.91E-01	−1.14E-03	9.54E-01	–
genus Bilophila	IA	IVW	0.98(0.93–1.03)	4.13E-01	5.08E-01	3.60E-03	8.54E-01	–
genus Ruminococcus1	IA	IVW	1.00(0.96–1.04)	9.66E-01	5.30E-01	5.55E-03	7.45E-01	–
family Streptococcaceae	IA	IVW	0.98(0.94–1.03)	4.14E-01	9.85E-01	−6.99E-03	6.83E-01	–
genus Prevotella7	IA	IVW	0.97(0.88–1.07)	5.75E-01	6.38E-01	4.25E-03	9.08E-01	–
genus Streptococcus	IA	IVW	0.98(0.94–1.03)	4.75E-01	9.60E-01	−5.30E-03	7.58E-01	–
genus Ruminococcus1	SAH	IVW	0.98(0.93–1.04)	5.10E-01	5.06E-01	3.77E-02	2.95E-01	–
genus Bilophila	SAH	IVW	1.00(0.93–1.07)	9.21E-01	2.47E-01	7.75E-03	8.75E-01	–
family Porphyromonadaceae	SAH	IVW	0.94(0.87–1.01)	1.02E-01	1.08E-01	−3.65E-02	4.31E-01	–
genus Fusicatenibacter	SAH	IVW	0.97(0.92–1.02)	2.53E-01	7.29E-01	−1.14E-02	7.21E-01	–
class Lentisphaeria	SAH	IVW	0.92(0.83–1.02)	1.12E-01	7.11E-01	4.06E-02	5.18E-01	–
order Victivallales	SAH	IVW	0.92(0.83–1.02)	1.12E-01	7.11E-01	4.06E-02	5.18E-01	–
genus Adlercreutzia	uIA	IVW	1.04(0.97–1.10)	2.68E-01	1.93E-01	−6.39E-02	3.31E-01	–
genus Intestinimonas	uIA	IVW	0.97(0.93–1.02)	2.10E-01	7.34E-01	4.30E-03	9.23E-01	–
genus Bilophila	uIA	IVW	1.01(0.97–1.05)	7.04E-01	9.79E-01	1.63E-02	7.06E-01	–
family Oxalobacteraceae	uIA	IVW	1.05(0.98–1.12)	1.70E-01	7.15E-01	−2.51E-02	7.14E-01	–
genus Victivallis	uIA	IVW	1.01(0.92–1.12)	7.78E-01	4.65E-01	−1.25E-01	2.12E-01	–

OR, odds ratio; CI, confidence interval; MRPRESSO, MR-Pleiotropy Residual Sum and Outlier; IA, intracranial aneurysms; SAH, subarachnoid hemorrhage; uIA, unruptured intracranial aneurysm; IVW, Inverse variance weighted.

### 5.5. Mediating Mendelian randomization analysis

A two-step Mendelian randomization study found that gut microbiota had a causal relationship with cerebrovascular disease, mediated by T2D and SBP (Table 5 and Supplementary Table S8). Specifically, T2D mediated the effects of genus Streptococcus on AS, AIS, and SVS, with mediating effects of 0.013, 0.014, and 0.027, accounting for 11.40%, 9.12%, and 7.42% of the total effects, respectively. On the other hand, SBP mediated the association between genus *Eubacterium brachy* group and AIS, with a mediating effect of −0.013, accounting for 17.63% of the total effect.

**Table 5:** The results of mediation Mendelian randomization analysis.

Exposure	Mediation factors	Outcome	β1	β2	β3	Mediating effect	The percentage of the mediating effect
genus Streptococcus	T2D	AS	0.11	0.15	0.09	0.014	12.73%
genus Streptococcus	T2D	AIS	0.15	0.15	0.10	0.015	10.00%
genus Streptococcus	T2D	SVS	0.37	0.15	0.19	0.029	7.84%
genus *Eubacterium brachy* group	SBP	AIS	−0.07	−0.37	0.03	−0.011	15.71%

T2D, type 2 diabetes; SBP, systolic blood pressure; AS, any stroke; AIS, any ischemic stroke; SVS, small vessel stroke.

## 6. Discussion

Cerebrovascular diseases are associated with high incidence, disability, and fatality rates. Many studies (Benakis et al., 2020; Zhu et al., 2020; Chidambaram et al., 2022) have demonstrated that the alteration of gut microbial composition *via* the microbiota-gut-brain axis (MGBA) facilitates the development and progress of these cerebrovascular diseases. The MGBA, which functions as a comprehensive communication network in the human body, has been found to interact significantly with various factors including brain structure, brain function, and nervous system diseases. Therefore, investigating the relationship between gut microbiota and cerebrovascular diseases can offer valuable insights for the effective prevention and treatment of these conditions. In this study, we conducted a Mendelian randomization analysis using GWAS summary statistics of gut microbiota and cerebrovascular diseases to evaluate the potential causal relationship.

The pathogenesis of intracranial aneurysms (IA), a cerebrovascular disease with significant health implications, is not yet fully understood. However, emerging evidence suggests that inflammation plays a crucial role in its development (Berge et al., 2016). Inflammation, in turn, is implicated in many diseases that are influenced by intestinal microbes (Zhao et al., 2021; Cai et al., 2022). Therefore, there is reason to believe that gut microbiota may impact the formation and progression of IA by modulating the body’s inflammatory response. Shikata et al. (2019) provided initial direct confirmation of this hypothesis by demonstrating that antibiotics could mitigate inflammation in cerebral arteries during IA formation, resulting in reduced IA incidence. In addition, another study (Kawabata et al., 2022) elucidated the relationship between gut microbiota imbalance and ruptured intracranial aneurysms, showing significant differences in gut microbiome characteristics between patients with uIA and patients with RA. And they concluded that Campylobacter could promote vascular remodeling and cell death of the cerebral artery wall by increasing inflammation-related cytokines, neutrophil-derived proteolysis, and oxidative stress (Kawabata et al., 2022). At the same time, it can finally lead to the rupture of IAs through the effects of hemodynamics and genetics (Kawabata et al., 2022). In addition to inflammation, metabolites of gut microbes may influence aneurysm rupture. Shikata et al. (2019) and Xu et al. (2023) found that taurine reduced the risk of aneurysm formation and rupture in mice, and taurine supplementation reversed aneurysm progression.

Similarly, stroke as a global health problem, already has a large number of studies have found that gut microbes by metabolic pathways and immune response in the steady state of the human body environment, affect the occurrence and development of stroke (Huang and Xia, 2021; Xu et al., 2021; Peh et al., 2022). Among them, atherosclerosis is considered to be an important mechanism of ischemic stroke. Gut microbiota can affect the development of atherosclerosis in three different ways: (GBD 2016 Causes of Death Collaborators, 2017) Gut microbiota can activate the immune system by affecting various immune cells, and macrophages can lead to increased proinflammatory cytokines and chemokines, thereby accelerating the formation of atherosclerotic plaques. For example, *Porphyromonas gingivalis* (Hayashi et al., 2011), Actinomycetes aggregobacter, *Chlamydia pneumoniae* (Blessing et al., 2001), and so on (An et al., 2017). Intestinal flora metabolism of cholesterol, fat and other foods affects the formation of atherosclerotic plaque (Vlacil et al., 2020; Tawk et al., 2021). Some metabolites produced by gut microbiota can promote the formation of atherosclerotic plaque by activating platelet activity. Among them, trimethylamine N-oxide (TMAO) pathway is considered to be the most direct pathway (Wang et al., 2011; Din et al., 2019). Clinical studies have shown that the increase of inflammation-related monocytes caused by elevated TMAO levels increases the risk of stroke and impairs the severity of stroke (Zhu et al., 2018, 2021). In addition, gut microbiota also produces metabolites that promote stroke recovery, among which short-chain fatty acids (SCFA) are one of the key molecules studied. Studies have shown that fecal transplantation containing higher SCFA levels or related bacteria can effectively alleviate neurological deficits and inflammation after stroke in aged male mice, and promote recovery after stroke in aged mice (Lee et al., 2020). Meanwhile, SCFA has been shown to promote post-stroke recovery by altering the recruitment of brain-resident immune cells in the brain (Sadler et al., 2020). Therefore, by reducing the level of TAMO and increasing the level of SCFA in the body, it is the goal of treating patients with ischemic stroke in the future.

However, current research on gut microbiota and cerebrovascular diseases remains extremely challenge. The diversity of gut microbiota is closely related to the environment, and the composition of gut microbiota is diverse in different regions. For example, one study reported that the gut microbiota of the Japanese population was quite different from that of other populations (Park et al., 2021). Thus, the present observational study may have specific limitations. In the future, we need to further expand the scope of our research to understand the onset and progression of other gut microbiota and cerebrovascular diseases.

The previous Mendelian randomization study (Meng et al., 2023) on gut microbes and ischaemic stroke employed a selection criterion of *p* < 5×10^−6^ and *p* < 5×10^−8^ for instrumental variables, which could have enhanced the persuasiveness of the findings. However, there were certain limitations in the study. Firstly, it did not explore the causal relationship between gut microbes and stroke and did not investigate the possibility of reverse causality. Additionally, the study did not correct for multiplicity, compromising the robustness of the results. However, there are some limitations to be considered when interpreting the results of this study. Firstly, subgroup analyses to explore the presence of nonlinear relationships could not be performed due to the use of summary statistics instead of raw data in the analysis. Secondly, in order to conduct sensitivity analyses and horizontal pleiotropy tests, it would be necessary to include more genetic variants as instrumental variables. Therefore, the SNPs utilized in the analysis did not meet the traditional GWAS significance threshold (*p* < 5 × 10^−8^). To address this issue, a Bonferroni correction was implemented to minimize the risk of false positives. Thirdly, the limitation of the exposure dataset, where the lowest taxonomic level was at the genus level, restricted further investigation into the causal relationship between gut microbiota and cerebrovascular disease at the species level. Lastly, it is important to note that although the majority of subjects in the GWAS meta-analysis of gut microbiota data were of European ancestry, confounding by population stratification is a possibility, and the results may not be fully generalizable to non-European populations.

## 7. Conclusion

This study provides new insights into the mechanisms of gut microbiota mediated cerebrovascular disease by presenting evidence of a potential causal relationship between gut microbiota and cerebrovascular disease. Moreover, systolic blood pressure and type 2 diabetes may mediate this process. To validate this finding, further longitudinal studies and trials could be conducted, which could potentially lead to the recommendation of more targeted probiotics in the prevention of IA and stroke.

## Data availability statement

The original contributions presented in the study are included in the article/Supplementary material, further inquiries can be directed to the corresponding author.

## Author contributions

HQ and FY: conceptualization, formal analysis, writing—original draft preparation, and writing—review and editing. HQ: methodology and data curation. HQ and PH: software and resources. XZ: validation and visualization. HQ, XZ, and FY: supervision. HQ, FY, PH, and XZ commented on the manuscript. All authors contributed to the article and approved the submitted version.

## Funding

The MEGASTROKE project received funding from sources specified at https://www.megastroke.org/acknowledgements.html.

## Conflict of interest

The authors declare that the research was conducted in the absence of any commercial or financial relationships that could be construed as a potential conflict of interest.

## Publisher’s note

All claims expressed in this article are solely those of the authors and do not necessarily represent those of their affiliated organizations, or those of the publisher, the editors and the reviewers. Any product that may be evaluated in this article, or claim that may be made by its manufacturer, is not guaranteed or endorsed by the publisher.

## Figures and Tables

**Table:** 

IS	ischemic stroke
GWAS	genome-wide association study
LAS	large artery stroke
SVS	small vessel stroke
CES	cardioembolic stroke
AS	any stroke
AIS	any ischemic stroke
IA	intracranial aneurysm
uIA	unruptured intracranial aneurysm
RA	ruptured intracranial aneurysm
SAH	subarachnoid hemorrhage
SBP	systolic blood pressure
T2D	type 2 diabetes
BMI	body mass index
IVW	inverse variance weighted
SNP	single nucleotide polymorphisms
MR	Mendelian randomization
IV	instrumental variables
TMAO	trimethylamine N-oxide
SCFA	short-chain fatty acids
MAF	minor allele frequency
MR-PRESSO	MR-Pleiotropy Residual Sum and Outlier
MGBA	microbiota-gut-brain axis
LD	linkage disequilibrium
